# Psychiatric manifestations of rare variation in medically actionable genes: a PheWAS approach

**DOI:** 10.1186/s12864-022-08600-x

**Published:** 2022-05-19

**Authors:** Yen-Chen A. Feng, Ian B. Stanaway, John J. Connolly, Joshua C. Denny, Yuan Luo, Chunhua Weng, Wei-Qi Wei, Scott T. Weiss, Elizabeth W. Karlson, Jordan W. Smoller

**Affiliations:** 1 Center for Genomic Medicine, Massachusetts General Hospital, Boston, MA USA; 2 Broad Institute of MIT and Harvard, 415 Main Street, Cambridge, MA USA; 3 Institute of Epidemiology and Preventive Medicine, National Taiwan University, Taipei, Taiwan; 4 Master of Public Health Program, National Taiwan University, Taipei, Taiwan; 5 Division of Nephrology, School of Medicine, Kidney Research Institute, University of Washington, Seattle, WA USA; 6 Center for Applied Genomics, The Children’s Hospital of Philadelphia, Philadelphia, PA USA; 7 Department of Biomedical Informatics, Vanderbilt University Medical Center, Nashville, TN USA; 8 Department of Medicine, Vanderbilt University Medical Center, Nashville, TN USA; 9 All of Us Research Program, National Institutes of Health, Besthesda, MD USA; 10 Department of Preventive Medicine, Northwestern University Feinberg School of Medicine, Chicago, IL USA; 11 Department of Biomedical Informatics, Columbia University Irving Medical Center, New York, NY USA; 12 Department of Medicine, Brigham and Women’s Hospital, Boston, MA USA; 13 Channing Division of Network Medicine, Brigham and Women’s Hospital, Boston, MA USA; 14 Division of Rheumatology, Inflammation and Immunity, Brigham and Women’s Hospital, Boston, MA USA; 15 Center for Precision Psychiatry, Massachusetts General Hospital, Boston, MA USA

**Keywords:** Genomic sequencing, Incidental findings, Rare variation, Psychiatric disorders

## Abstract

**Background:**

As genomic sequencing moves closer to clinical implementation, there has been an increasing acceptance of returning incidental findings to research participants and patients for mutations in highly penetrant, medically actionable genes. A curated list of genes has been recommended by the American College of Medical Genetics and Genomics (ACMG) for return of incidental findings. However, the pleiotropic effects of these genes are not fully known. Such effects could complicate genetic counseling when returning incidental findings. In particular, there has been no systematic evaluation of psychiatric manifestations associated with rare variation in these genes.

**Results:**

Here, we leveraged a targeted sequence panel and real-world electronic health records from the eMERGE network to assess the burden of rare variation in the ACMG-56 genes and two psychiatric-associated genes (*CACNA1C  and TCF4*) across common mental health conditions in 15,181 individuals of European descent. As a positive control, we showed that this approach replicated the established association between rare mutations in *LDLR* and hypercholesterolemia with no visible inflation from population stratification. However, we did not identify any genes significantly enriched with rare deleterious variants that confer risk for common psychiatric disorders after correction for multiple testing. Suggestive associations were observed between depression and rare coding variation in *PTEN* (*P* = 1.5 × 10^–4^), *LDLR* (*P* = 3.6 × 10^–4^), and *CACNA1S* (*P* = 5.8 × 10^–4^). We also observed nominal associations between rare variants in *KCNQ1* and substance use disorders (*P* = 2.4 × 10^–4^), and *APOB* and tobacco use disorder (*P* = 1.1 × 10^–3^).

**Conclusions:**

Our results do not support an association between psychiatric disorders and incidental findings in medically actionable gene mutations, but power was limited with the available sample sizes. Given the phenotypic and genetic complexity of psychiatric phenotypes, future work will require a much larger sequencing dataset to determine whether incidental findings in these genes have implications for risk of psychopathology.

**Supplementary Information:**

The online version contains supplementary material available at 10.1186/s12864-022-08600-x.

## Background

Rapid advances in sequencing technologies accompanied by progressive cost reductions are fueling efforts to incorporate genetic data into medical practice. Genomic medicine offers important opportunities to improve diagnosis and treatment of both complex and Mendelian diseases [1, 2]. At the same time, clinical applications of exome and genome sequencing can produce incidental or secondary findings that are not the primary target of genetic evaluation but have implications for genetic counseling and patient care. The American College of Medical Genetics and Genomics (ACMG) has recommended return of results (RoR) for an expanding list of genes (56 genes in v1.0 and 59 genes in v2.0) in which pathogenic coding variants are highly penetrant and medically actionable [3, 4]. Current RoR guidelines, however, largely focus on a single established phenotype-genotype association, while genetic analyses have revealed that “pleiotropy”—in which a variant or gene affects multiple phenotypes—is ubiquitous across the human genome, including some of the genes recommended by ACMG for incidental findings (referred to as “ACMG genes” in the rest of this paper) [5, 6].

Psychiatric disorders are common, complex phenotypes affecting more than a billion people worldwide. Genome-wide association studies (GWAS) have found extensive pleiotropy of both common and rare variants across mental illnesses and other diseases (e.g., cardiovascular and immune-related disorders) [7–10]. Although psychiatric disorders are highly polygenic reflecting the influence of hundreds to thousands of common genetic loci [11–13], recent large-scale sequencing studies of these disorders based on tens of thousands of individuals have begun to identify individual genes harboring an excess of de novo or rare disruptive variants in cases compared to unaffected individuals [14–16]. To date, however, there has not been a systematic evaluation of the spectrum of psychiatric manifestations associated with rare variation in the ACMG genes or other genes where specific variants have been robustly shown to influence the risk of major psychiatric disorders. Studying these relationships is important to improve our understanding of the nature of pleiotropy among the ACMG genes, inform RoR guidelines for incidental findings regarding psychiatric disorders, and disentangle its heterogeneous genetic architecture.

Here, we leverage data from the Electronic Medical Records and Genomics (eMERGE) consortium that includes real-world phenome-wide medical data and deep sequencing of the ACMG-56 genes (v1.0; Table S1) [17, 18], as well as two genes previously implicated in major psychiatric traits (*CACNA1C* and *TCF4*) [19, 20] to examine whether rare variants in these genes are associated with mental illnesses for 15,181 individuals of European descent. Through a phenome-wide association study (PheWAS) approach [21], we performed gene-based burden tests across 37 curated psychiatric disorders with adequate sample size and discuss findings and limitations for future research.

## Results

### Overview of the targeted sequence and phenotype data

To explore the nature of pleiotropy of rare variation in the ACMG-56 genes and two additional genes (*CACNA1C* and *TCF4*) on psychiatric manifestations, we used a deep, targeted sequence panel from the eMERGE network (eMERGEseq). While showing a clear multi-ancestry structure, the panel consisted of predominantly individuals of European (EUR) descent (Figure S1), to which we limited the subsequent rare-variant analysis for power considerations. After quality control procedures (Methods), the data comprised 15,181 EUR individuals with complete phenotype information from electronic health records (EHR) and ~ 13,000 variants at minor allele frequency (MAF) < 1% in the 58 genes. We then mapped the International Classification of Disease diagnostic codes (ICD-9-CM and ICD-10-CM) to PheWAS codes (PheCodes) [22, 23] and defined eligible cases for a specific PheCode as having at least two codes on two separate calendar dates (Table S2).

For our rare-variant PheWAS of mental disorders, we curated 37 psychiatric symptoms and disorders with a minimum in-sample prevalence of 0.5% (Ncase ≥ 75) (Table S3). As prevalence of these conditions largely reflected disease frequency in the population, many psychiatric disorders were filtered out, including schizophrenia (prev = 0.3%; 44 cases). Among the curated list, conditions with the highest prevalence included tobacco use disorder (prev = 18.4%; 2783 cases) and depression (prev = 17.1%; 2590 cases), and lowest for personality disorder (0.6–0.7%; 113 cases) and psychosis (0.6%; 90 cases). Other major psychiatric disorders included autism (3.7%; 572 cases), attention-deficit/hyperactivity disorder (7.6%; 1164 cases), and bipolar disorder (2.6%; 397 cases). The majority of the 37 psychiatric conditions were significantly, positively correlated (Spearman’s correlation, range: -0.09–0.57; median: 0.05; Table S4; Figure S2). In contrast, phenotypic correlations between the evaluated psychiatric conditions and other phenotypes were much weaker (Table S5).

### Replication of a known association with no visible signs of population stratification

Singe variant association analysis of two selected complex traits (obesity [Ncase = 3,521; PheCode: 278.1] and essential hypertension [Ncase = 6,972; PheCode: 401.1]; MAF > 0.1%) suggested there was no signs of inflation in test statistics due to population stratification or other confounding factors (λ_GC_ ~ 1; Figure S3). As a positive control analysis, we conducted a PheWAS of 966 available PheCodes with at least 75 cases (Table S2) against rare variants in the LDL-receptor (*LDLR*) gene, in which more than 3,000 mutations, predominantly missense variants, have been described to cause familial hypercholesterolemia (FH) (ClinVar) [24–26]. Unsurprisingly, our results recapitulated this known association, showing a clear and significant enrichment of rare deleterious variation in people with hypercholesterolemia, specifically for damaging missense variants (*p-*value = 1.1 × 10^–9^). As a negative control, synonymous variation, a presumably neutral class of variants, showed no association signals with any of the tested traits (Figure S4).

### Burden of rare variation in ACMG-56 genes in psychiatric disorders

To assess whether individuals affected with psychiatric disorders carried an excess of rare variants in the ACMG-56 genes, we considered five categories of qualifying variants to calculate an aggregate burden (all rare variants with MAF < 1%, all non-synonymous variants, all rare PTVs, all rare damaging missense variants, and all rare PTVs *and* damaging missense variants) and carried out the analysis using Firth’s logistic regression and Fisher’s Exact test (Methods).

**Fig. 1:**
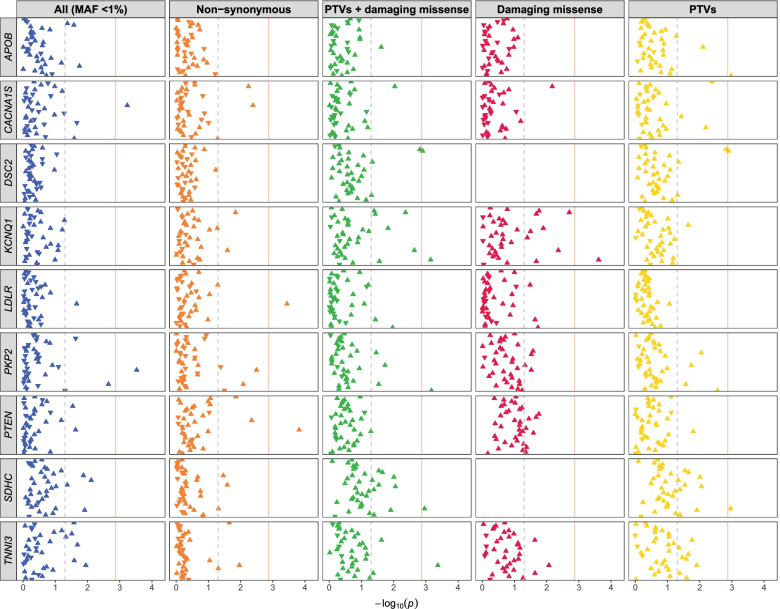
PheWAS of rare variation in the selected ACMG-56 genes with psychiatric disorders. Show here are the PheWAS results of 9 genes listed in Table 1 with suggested significance. For each gene, five-panel results for all 37 tested psychiatric conditions are shown, separately for (1) all variants with MAF < 1%, (2) all non-synonymous variants, (3) all PTVs and damaging missense variants combined, (4) damaging missense variants, and (5) PTVs (from left to right). On the x-axis shows the -log10(p-value) of the burden tests. Each triangle represents a disorder, with an upright triangle indicating the gene is associated with an increased risk (OR > 1) of the disorder and an inverted triangle indicating a decreased risk (OR < 1). Genes with no qualifying variants present among the study participants were not tested and are left vacant in the figure. The vertical dotted grey line for each individual PheWAS signifies the nominal significance level of 0.05, and the vertical red solid line represents the Bonferroni-corrected significance (0.05/37 = 1.35 × 10–3). No association surpassed the study-wide significance at FDR < 0.05. The full set of PheWAS results for the ACMG-56 genes can be found in Table S6 & Figure S5

Among the tested gene-disorder relationships, we did not identify any study-wide significant gene associations (FDR < 0.05), with only 11 pairs reaching individual-PheWAS significance (Bonferroni-adjusted significance threshold of 1.35 × 10^–3^; Table 1; Table S6; Fig. 1; Figure S5). Results were generally comparable between Firth’s logistic regression and Fisher’s Exact test, with slightly stronger signals in the former (Table S6). Some of these suggestive associations included major depressive disorder and *PTEN*, a tumor suppressor gene (burden of non-synonymous variants: OR = 9.15, Firth’s *p*-value = 1.5 × 10^–4^); substance addiction and disorders and *KCNQ1*, which encodes a potassium channel protein (burden of damaging missense variants: OR = 14.63, *p*-value = 2.4 × 10^–4^), and depression and *CACNA1S*, a calcium channel gene (burden of all rare variants: OR = 1.50, *p*-value = 5.8 × 10^–4^; Table 1). A few genes causing rare or Mendelian heart conditions were also implicated, such as *LDLR* (burden of nonsynonymous variants in major depressive disorder: OR = 2.10, *p*-value = 3.6 × 10^–4^) and *APOB* (burden of PTVs in Tobacco use disorder: OR = 12.99, *p*-value = 1.1 × 10^–3^), both linked to familial hypercholesterolemia (Table S1). The associated phenotypes among the suggestive associations are broader PheCode terms involving combinations of related disorders and symptoms.

**Table 1:** Suggestive associations of rare variant burden in the ACMG-56 genes with psychiatric disorders

Phenotype	PheCode	Gene	Consequence	Ncase	Ncontrol	Ncase carrier	Ncontrol carrier	OR	95% CI	*P*-value
Major depressive disorder	296.22	*PTEN*	Non-synonymous	2312	12,869	8	8	9.15	(3.03, 27.6)	1.5 × 10^–4^
Substance addiction and disorders	316	*KCNQ1*	Damaging missense	446	14,735	4	10	14.63	(4.31, 49.6)	2.4 × 10^–4^
Mental retardation	315.3	*PKP2*	All	123	15,058	5	168	8.32	(3.24, 21.4)	3.0 × 10^–4^
Major depressive disorder	296.22	*LDLR*	Non-synonymous	2312	12,869	35	105	2.10	(1.42, 3.11)	3.6 × 10^–4^
Other mental disorder	306	*TNNI3*	PTV + damaging mis	1759	13,422	3	0	65.85	(1.90, 2279)	4.2 × 10^–4^
Depression	296.2	*CACNA1S*	All	2590	12,591	100	327	1.50	(1.20, 1.88)	5.8 × 10^–4^
Tobacco use disorder	318	*PKP2*	PTV + damaging mis	2783	12,398	5	2	15.98	(2.41, 106)	6.7 × 10^–4^
Substance addiction and disorders	316	*KCNQ1*	PTV + damaging mis	446	14,735	4	14	10.56	(3.36, 33.2)	7.1 × 10^–4^
Tobacco use disorder	318	*APOB*	PTV	2783	12,398	6	2	12.99	(2.19, 76.9)	1.1 × 10^–3^
Somatoform disorder	303.4	*SDHC*	PTV	130	15,051	1	1	160.70	(8.64, 2987)	1.1 × 10^–3^
Alcoholism	317.1	*DSC2*	PTV	334	14,847	2	3	34.33	(4.68, 252)	1.3 × 10^–3^

For each gene, a PheWAS was performed to assess the burden of rare variation (MAF < 1%) in 37 mental illnesses (Table S3) using Firth’s logistic regression, adjusting for age, sex, sites, and the first 10 PCs. This analysis was done separately for five groups of qualifying variants (all variants with MAF < 1%, non-synonymous variants, damaging missense variants, protein-truncating variants or PTVs, and PTVs plus damaging missense variants). Shown in the table are suggestive gene-disorder associations at a Bonferroni-corrected significance of 0.05/37 = 1.35 × 10^–3^ in an individual PheWAS. “Carrier” is defined as individuals who carried at least one copy of the qualifying variant in the gene. No genes achieved study-wide statistical significance at FDR < 0.05 accounting for all tests. The full set of gene-disorder associations are depicted in Fig. 1. *OR* odds ratio, *CI* confidence interval

Scanning across the associations, we noted an overall low count of qualifying variants, with several genes lacking qualifying PTVs and damaging missense variants among study participants (Fig. 1; Figure S5), and many PheCodes with no mutation carriers among either cases or controls (Table S6). While Firth’s logistic regression applied bias correction to rare events to control for type I error, the possibility of inflation might not be ruled out due to these extremely low counts. These results indicate a general lack of power for gene-based burden tests to detect significant genes for mental disorders given the current sample size.

### No enrichment of rare variants in two genes previously associated with major mental disorders

For *CACNA1C and TCF4*, two genes that have been repeatedly linked to major psychiatric disorders from GWAS and a few rare variant association studies, we studied the role of deep-sequenced rare variation across a spectrum of mental illnesses. In brief, *CACNA1C* is a voltage-gated calcium channel gene that was found to associate with schizophrenia, bipolar disorder, Timothy syndrome, and autism, while *TCF4*, a transcription factor gene, has been implicated in schizophrenia, bipolar disorder, major depressive disorder, post-traumatic stress disorder, and Pitts Hopkins syndrome [12, 13, 19, 20, 27–33] (Methods).

**Fig. 2:**
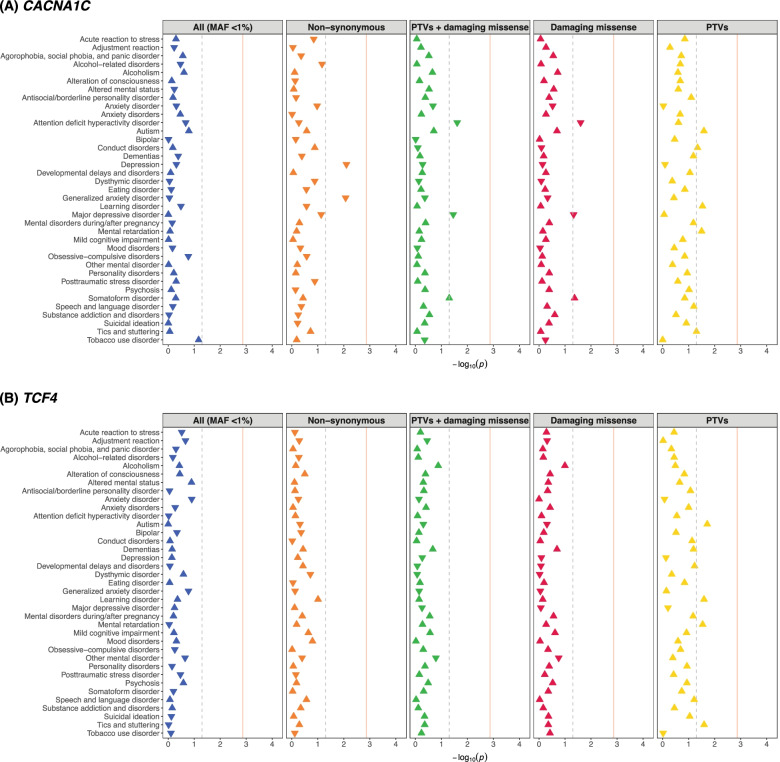
PheWAS of rare variation in *CACNA1C* (**A**) and *TCF4* (**B**) with 37 psychiatric disorders. Findings from burden tests that assess the enrichment of each variant category in psychiatric disorders are depicted; x-axis shows the -log10(p-value) across all 37 psychiatric conditions on the y-axis. An upright triangle indicating a risk-increasing effect (OR > 1) of the tested variant class and an inverted triangle indicating a risk-decreasing association (OR < 1). The vertical dotted grey line represents the nominal significance level of 0.05, and the vertical red solid line represents the Bonferroni-corrected significance (1.35 × 10–3). No association surpassed the study-wide significance at FDR < 0.05

When evaluating the burden of rare variation in *CACNA1C* and *TCF4*, we did not detect any suggestive or study-wide significant associations with the tested psychiatric conditions (Tables S7-8; Fig. 2). Note that our study of 15,181 individuals included a limited number of individuals affected with major psychiatric disorders from EHR (e.g., bipolar disorder, 397 cases; autism, 572 cases; Table S3), raising the possibility of Type II error.

### No evidence of the rare-variant burden stratifying by age or sex

As the age range in our sample is broad and the proportion of females differs by PheCodes (Table S3), we explored possible effect modifications by age and sex on the association results. Specifically, we performed secondary PheWAS analyses involving an interaction of age or sex with the count of qualifying rare variants for each gene. Nonetheless, similar to the primary findings, we did not observe any significant interaction at study-wide significance (FDR < 0.05) across the 37 psychiatric PheCodes and 58 genes for all five functional groupings of rare variants (Tables S9-10). Thus, there was no statistical evidence that the burden of rare variation on these psychiatric conditions varied by age or sex in the present study.

### No significant findings from gene-set-based PheWAS grouped by biological domains

Considering that rare variants with the same predicted functional consequence or involved in the same biological mechanism may act together to influence disease risk, we took a more systematic approach that grouped qualifying variants into gene sets and tested for enrichment in patients affected with the evaluated psychiatric conditions versus control individuals. Specifically, we looked at all 58 genes in aggregate (Table S11) and separately for three gene sets each associated with the most significant Gene Ontology (GO) term for “biological process” [34 genes, circulatory system development (GO:0072359); Table S12], “cellular component” [16 genes, contractile fiber (GO:0043292); Table S13], or “molecular function” [25 genes, protein-containing complex binding (GO:0044877); Table S14]. Nonetheless, as with single-gene PheWAS results, we did not identify any significant gene-set associations after multiple testing correction (Tables S11, S15,16,17).

### Comparison with gene associations from the UK Biobank (UKB)

To seek potential validation from publicly available resources, we then compared our results to individual gene associations reported in the UK Biobank (UKB) involving 281,852 individuals of European ancestry (available on GeneBass: https://genebass.org/; [34]. Notably, UKB is a population-based cohort that appears to have a healthy volunteer selection bias [35].

For the 16 psychiatric PheCodes that could be mapped to a UKB definition, we observed that the prevalence was overall much lower in UKB than in our eMERGE study (Table S18; e.g., anxiety disorders, eMERGE: 17% vs. UKB: 5%). Despite differences in sample ascertainment, quality control, and analysis pipelines, both our eMERGE results and those reported in GeneBass identified no genes significantly associated with the tested psychiatric phenotypes at FDR < 0.05 (Table S19). Additionally, association signals in the UKB-GeneBass data were weaker than in eMERGE (Table S20).

## Discussion

Incidental findings in the ACMG genes have been recommended for RoR but no studies to our knowledge have systematically examined whether rare variation in these genes is associated with psychiatric manifestations in deeply sequenced samples. To the extent that such variants affect psychopathology, they could have relevance for genetic counseling when returning results for medically actionable genes. In the present study, we explored this issue using a targeted sequence panel and real-world EHR data from the eMERGE consortium through a PheWAS approach. In addition, we took the opportunity of interrogating two additional genes (*CACNA1C* and *TCF4*) widely implicated in major psychiatric disorders that were also included in the targeted panel. Despite deep sequencing of 15,181 individuals, our study did not identify any significant genes in which rare deleterious variants were enriched in patients with mental disorders after study-wide multiple testing correction. There was also no statistical evidence that age or sex influenced the pattern of the rare-variant burden on these psychiatric conditions, nor did we identify significant gene sets across the 58 genes in which rare variation involved in the same biological process collectively affected disease risk. When compared with publicly available gene associations reported from UKB in the GeneBass resource [34], we observed similar null results while association signals in the UKB-GeneBass database were generally weaker than in our study.

One major limitation is the limited statistical power for detecting rare variant associations given the relatively small number of cases from our EHR-based study (Table S3). Due to the low population prevalence, modest SNP-heritability, and complex genetic architecture of psychiatric disorders, GWAS of common genetic variation have shown that hundreds of thousands of affected individuals are often required to detect genetic risk factors underlying mental illnesses, such as through meta-analysis of case-enriched cohorts [11–13]. For analysis of rare variation, sample size requirements may be even larger. Recently, two whole-exome studies of schizophrenia and bipolar, each with over 10,000 cases and 10,000 controls, began to identify significant risk genes with rare PTVs and damaging missense variants conferring a strong harmful effect on the disease and shed light on the underlying biology of the contribution of rare disruptive variants relative to common variants [14, 15]. Notably, among findings from these large exome-based studies, *CACNA1C* and *TCF4* did not emerge as top signals with significant enrichment.

Related to sample size, we note several caveats to our study. First, the limited number of available individuals for rare variant analysis made it challenging to exclude the possibility of false positives, even after multiple testing correction. Some of our top results had unusually large effect sizes that might be artifacts (Table 1; Tables S6,7,8). Second, while we initially intended to retain as many ancestries as possible in the study, the variant count (per gene) by sample matrix was extremely sparse for each of the smaller set of non-EUR individuals to power a multi-ancestry analysis. Further, in the burden analysis, we grouped variants according to algorithm-predicted functional consequences and deleteriousness. Because of the scarcity of the aggregated variants, we did not apply additional filters on qualifying variants to increase the specificity of the predicted deleteriousness, such as prioritizing variants classified by ClinVar as likely pathogenic or pathogenic [26], using allele frequency information in the Genome Aggregation Database (gnomAD) [36] as the control population for filtering, or applying the LOFTEE filter [36] to separate high-confidence PTVs from annotation artifacts. Future larger-scale studies could incorporate these considerations to optimize the signal-to-noise ratio. Relatedly, in our analyses we did not have the power to examine gene-disorder relationships assuming recessive inheritance, for which both copies of the gene must harbor the qualifying variant, or a compound heterozygous state, where two recessive alleles are at different locations of the gene. While current evidence of the genetic underpinnings of psychiatric disorders primarily points to a complex and polygenic architecture that does not follow a simple Mendelian model, ongoing data collection from several large-scale sequencing studies could further elucidate the mode of inheritance and penetrance regarding rare deleterious variation [14–16]. Lastly, we could not rule out that some of the gene associations from PheWAS (though not significant) are due to confounding or comorbidity with other phenotypes for a given psychiatric trait, which may complicate the interpretation of results. To account for those, we would need the complete comorbidity profiles and medication use among study subjects for each specific psychiatric diagnosis, ideally in a pre-specified time window to establish a clear temporality between these variables. Future larger-scale PheWAS-based studies could address these limitations.

Although we did not detect study-wide significant gene signals, suggestive associations showed that some of the ACMG-56 genes harbor an excess of rare deleterious variants in people with psychiatric conditions compared to controls (Table 1). Most of these associations have not been reported elsewhere and should be interpreted with caution given the caveats described above. It is interesting, nonetheless, that among the suggestive burden of rare variants are two channel genes, *KCNQ1* and *CACNA1S,* associated with substance use disorders and depression, respectively (Table 1), which involve in the formation and function of potassium and calcium channels that are broadly implicated in brain-related disorders [19, 37]. A SNP association in *KCNQ1* with alcohol dependence was previously reported from a GWAS of European Americans [38], and the *CACNA1S* gene region, along with other calcium channel genes, was associated with major psychiatric disorders from a GWAS meta-analysis of schizophrenia, bipolar disorder, major depressive disorder, autism, and attention-deficit/hyperactivity disorder [28]. While these suggestive associations did not appear as strong in the UKB-GeneBass database, we note that UKB may not be a well-powered resource for studying rare genetic variation and mental disorders as the whole-cohort disease prevalence for psychiatric-related conditions in UKB tends to be lower than in our real-world, EHR-based data (Table S18). Overall, these observations indicate the possibility of pleiotropy of the tested genes beyond the established link to rare medical conditions recommended for RoR (Table S1) but require further validation in independent samples ideally involving case-enriched cohorts.

## Conclusion

Return of incidental findings from clinical sequencing will become an increasingly important issue as genomic data generation and technology development advance into the next decades. The current study illustrates the opportunities and challenges of leveraging deep sequence data to identify incidental findings from medically actionable genes associated with psychiatric disorders, which exhibit a high degree of phenotypic and genetic complexity. Nonetheless, with the increasing availability of large-scale genomic sequencing efforts expanding to diverse populations (e.g., the *All of Us* Research Program [39], the incorporation of genomic data into medical practice, and the expanding list of genes with actionable mutations, characterizing the phenotypic spectrum of actionable mutations will have increasing implications for genomic medicine and genetic counseling.

## Methods

### Targeted sequence data from the eMERGE network

The eMERGE network is a national network that links electronic health records (EHR) and DNA sequence data of individuals for large-scale genetic and precision medicine research across 11 participating sites (Children’s Hospital of Philadelphia, Cincinnati Children’s Hospital Medical Center, Columbia University, Icahn School of Medicine at Mt. Sinai, Mass General Brigham [formerly Partners Healthcare], Mayo Clinic, Northwestern University, University of Alabama at Birmingham, University of Washington-Kaiser Permanente, and Vanderbilt University Medical Center) [17, 40]. Informed consent from adult participants and signed parental permission for participants under age 18 were obtained from each sample collection site under the respective Institutional Review Board (IRB)-approved protocols.

The eMERGEseq platform is a targeted sequence panel designed within the network including a selected set of genes for the purpose of returning results of pathogenic variants to participants as well as providing a resource for genetic research. The eMERGEseq panel comprised 109 genes and 1,551 single-nucleotide variants (SNVs) for 24,956 individuals, randomly selected from biorepositories at each eMERGE site. Details of informed consent, sample handling, DNA extraction, and sequencing were also described previously [40]. DNA from blood samples were extracted at individual eMERGE sites and sent to Baylor College of Medicine Human Genome Sequencing Center (HGSC) and the Broad Institute for sequencing. IRB approval for the two sequencing centers deferred consent to the participating sites (Mass General Brigham and Baylor College of Medicine). Specifically, genes in the panel included 56 genes with known pathogenic variants from the ACMG guidelines v1.0 [3] (Table S1) and 53 additional genes nominated from each eMERGE site based on established associations with specific disease areas. Among them, 2 genes (*CACNA1C* and *TCF4*) were nominated for their consistent links to major psychiatric disorders: *CACNA1C* encodes a voltage-gated calcium channel subunit and growing evidence has suggested that common single variants polymorphisms (SNPs) in this gene increase the risk of schizophrenia, bipolar disorder, while rare gain-of-function mutations lead to Timothy syndrome that features autism; some of the implicated SNPs also show a pleiotropic effect across psychiatric traits [12, 13, 19, 27–29]. *TCF4*, a transcription factor gene, is involved in the initiation of neuronal differentiation with common polymorphisms implicated in GWAS of schizophrenia, bipolar disorder, major depressive disorder, and post-traumatic stress disorder [28, 32, 33], and disruptive mutations known to cause Pitts-Hopkins syndrome characterized by intellectual disability and autistic behavior [20, 30, 31].

The sequenced dataset was shared with each site for linking with EHR for genetic analysis.

### Sequence data quality control

To conduct PheWAS of genes in the targeted panel, we performed quality control procedures to retain a list of high-quality variants and samples. Raw sequence of the eMERGEseq dataset consisted of 59,141 SNPs and 3,358 insertion/deletions (indels) on human genome GRCh37 build, with an average sequence depth above 500X across individuals. Variants were first left-normalized and multiallelic sites were split into biallelic forms. Variants that failed the GATK VQSR (Variant Quality Score Recalibration) metric and those lying outside of low complexity regions [41] were removed. Genotypes were set to missing for homozygous reference genotype calls with an allelic balance (AB) > 0.1, heterozygous calls with AB < 0.25 or AB > 0.75, homozygous variant genotype calls with AB < 0.9, and those with GQ < 20 or DP < 10. Samples with a low call rate (< 0.975) and outliers of transition/transversion ratio, heterozygous/homozygous ratio, or insertion/deletion ratio (> 3SD from the mean) were discarded. Next, we performed principal component analyses (PCA) to identify ancestral populations for study participants and used Random Forest Classifier with 1000 Genomes phase 3 data [42] as the training set to assign ancestry to each individual. With a prediction probability > 0.8, 16,641 participants were classified as European (EUR), 3,612 as African (AFR), 1,455 as Admixed/Latin American (AMR), 1,093 as East Asian (EAS), 271 as South Asian (SAS), and the remaining individuals with an unclassified ancestry (Figure S1). Despite the small number of independent markers in the targeted panel, PC plots showed clear separation of each ancestral population and results were comparable with that inferred by our internal genome-wide array data. Focusing on the EUR subset with an adequate sample size for power considerations, we then removed individuals with mismatched reported and genetic sex, one from each pair of related individuals, and excluded variants that were monomorphic, had a call rate < 0.95, or a Hardy–Weinberg Equilibrium (HWE) test *p*-value < 1 $\times$ 10^–6^. Finally, we removed a small proportion of common genetic variants (MAF > 0.01), yielding a QC’ed dataset of 16,512 individuals of EUR descent and 33,565 low-frequency and rare variants (MAF < 0.01). QC procedures were performed using bcftools [43] and PLINK (v1.90b3.32) [44].

### Variant annotation

Annotation of variants was performed with SnpEff [45] for human genome assemble GRCh37. Based on the predicted consequences, we defined three functional classes of coding variants: protein-truncating variant (PTV) (“disruptive_inframe_insertion”, “disruptive_inframe_deletion”, “frameshift_variant”, “splice_acceptor_variant”, “splice_donor_variant”, “stop_gained”, “feature_ablation”, or “exon_loss_variant”), missense variants (“inframe_insertion”, “inframe_deletion”, “missense_variant”, “splice_region_variant”, “stop_lost”, “start_lost”, “coding_sequence_variant”), and non-synonymous variants (PTVs and missense variants combined). Further, to discriminate likely deleterious missense variants from benign missense variants, we applied seven in silico missense deleteriousness predictors (SIFT, PolyPhen2 with HDIV training set, PolyPhen2 with HVAR training set, LRT, MutationTaster, MutationAssessor, and PROVEAN [46] to identify a subset of highly damaging missense variants that were predicted as deleterious by all seven algorithms.

### Phenome-wide association study (PheWAS) of psychiatric manifestations

EHR data for the 24,956 individuals were extracted and cleaned from each contributing site in the eMERGE network, which contains a combination of International Classification of Disease version 9 and 10 (ICD-9-CM and ICD-10-CM) diagnostic codes. Both ICD-9 and ICD-10 codes were mapped to PheWAS codes (PheCodes) [22, 23] for genetic analysis. Combined with the EUR subset of the eMERGEseq data, this resulted in 15,181 individuals and 1,858 PheCodes (age range: 6–100; median age: 62; 54.5% women). We defined eligible cases as having at least two instances of the same PheCode on two different calendar dates in the EHR (Table S2). We then curated a subset of 37 PheCodes that represent common psychiatric phenotypes with a minimum case count of 75 (equivalent to an in-sample prevalence of 0.5%; Table S3), with phenotypic correlations ranging from -0.09 to 0.57 (Table S4; Figure S2). For our PheWAS analysis of rare genetic variation and psychiatric manifestations, we focused on the ACMG-56 genes and the 2 additional genes (*CACNA1C* and *TCF4*) widely implicated in mental illnesses and tested the association under a burden analysis framework using the PheWAS R package [47]. Burden score for each individual was calculated as the sum of the aggregated qualifying variants observed in a given gene. We considered five categories of qualifying variants for analysis, including all rare variants (MAF < 0.01; total ~ 13,000 variants in the 58 genes) and four functional coding annotations: all rare non-synonymous variants, all rare PTVs, all rare damaging missense variants, and all rare PTVs *and* damaging missense variants. For each of the 37 PheCodes, a Firth’s logistic regression was implemented by regressing case–control status against variant count of a certain category in a given gene, adjusting for age, sex, sites, and the first 10 principal components (PCs). Firth’s method uses penalized maximum likelihood estimation in logistic regression and has been shown to yield good calibration and correct bias in association tests for low frequency and rare variants with unbalanced care-control ratios and complete separation [48–50]. A two-sided Fisher’s Exact test *p*-value was also calculated using the carrier counts among the cases and controls, defined as the number of individuals carrying at least one qualifying variant (i.e., a dominance test; Tables S5,6,7). We calculated a Bonferroni-corrected significance level (0.05/37 = 1.35 × 10^–3^) for individual PheWAS to detect suggestive associations and defined a study-wide significant enrichment at a false discovery rate (FDR) < 0.05 to account for multiple testing of all 10,730 burden tests (37 PheCodes, 58 genes, and 5 variant filters). Analyses were conducted using the R programming language (version 3.5).

### Secondary PheWAS analysis: interaction with age or sex

In addition to the primary PheWAS analysis, we performed secondary analyses to examine the influence of age or sex on the association results for the evaluated psychiatric conditions. For each of the 37 PheCodes, we performed Firth’s logistic regression following the same model as described in the primary analysis but added an interaction term between age or sex with the variant count of a certain category in a given gene. This involved another set of 10,730 burden tests for the age- and sex-interaction analysis separately, and we considered study-wide significant interactions at FDR < 0.05 accounting for all tests.

### Secondary PheWAS analysis: gene sets grouped by functional consequence or biological mechanism across all 58 genes

To evaluate whether rare variation functioning in a similar biological manner in distinct genes could collectively affect risk of each psychiatric condition, we analyzed the relationship between the 37 psychiatric PheCodes and the aggregate count of qualifying variants across the 58 genes (ACMG56, *CACNA1C* and *TCF4*). Additionally, we performed Gene Ontology (GO) analysis to identify significant GO terms associated with the 58 genes using over-representation tests [51]. Qualifying variants in a subset of the genes involved in the most significant GO term for each of the three independent domains (“biological process”, “cellular component”, and “molecular function”) were aggregated and tested for enrichment in affected individuals versus controls. Similar to the primary analysis, the gene-set PheWAS analysis was done separately for the five variant annotation categories using Firth’s logistic regression adjusting for age, sex, sites, and the first 10 PCs.

### Comparison with gene associations from the UK Biobank (UKB)

The UK Biobank (UKB) is a 500,000-person population-based cohort that includes a wide variety of phenotypes with genetic data also available for each individual [52]. Unlike our EHR-based study, phenotypes in UKB were measured through primarily self-report, with linkage to participants’ hospitalization records (International Classification of Diseases, ICD codes), primary care data, etc. Based on the whole-exome data released from UKB, efforts have been conducted to investigate the phenome-wise gene burden of deleterious variants. A large resource, GeneBass (Gene-Biobank Association Summary Statistics, https://genebass.org/), has been built that made available to the public gene-burden results from exome-sequence analysis of 281,852 European-descent individuals across 3,817 traits in the UKB [34]. To compare our results with those from UKB-GeneBass, we extracted association statistics of the ACMG genes, *CACNA1C*, and *TCF4* in GeneBass with UKB phenotypes that can be mapped to the 37 psychiatric PheCodes in eMERGE. When multiple phenotype definitions in UKB-GeneBass can be matched to a particular PheCode in eMERGE, we prioritized those measured for the whole cohort rather than in subsets whenever possible and chose the definition with the largest number of diseased individuals for comparison. GeneBass includes three sets of gene-level tests including burden test, SKAT, and SKAT-O implemented using the SAIGE-GENE mixed-model framework [53]. Details of variant annotation also differed to some extent between GeneBass and our pipeline [34]. For the purpose of comparison, we focused on burden test results from GeneBass for “pLoF” (analogous to PTV) and “missense” variants separately.

## Supplementary Information

**Additional file 1:** **Figure S1.** Ancestry assignment of study individuals using principal component analyses (PCA). **Figure S2.** Phenotypic correlation heatmap among the 37 curated PheCodes of common psychiatric phenotypes. **Figure S3.** No inflation in λGC from single variant association analysis for two selected complex traits. **Figure S4.** Phenome-wide burden of rare variation in LDLR recapitulated known association with hypercholesterolemia. **Figure S5.** PheWAS of rare variation in the ACMG-56 genes with psychiatric disorders

**Additional file 2: Table S1.  **A list of 56 genes and the associated medical conditions recommended by ACMG for return of results. **Table S2.** The full list of the extracted PheCodes and the corresponding prevalences among the 15,181 individuals analyzed in the study. **Table S3.** The 37 PheCodes of psychiatric disorders with at least 75 cases selected for burden analysis. **Table S4.** Pairwise phenotypic correlations between the 37 psychiatric phenotypes. **Table S5.** Pairwise phenotypic correlations between the 37 psychiatric phenotypes and all 966 phenotypes with at least 75 cases. **Table S6.** PheWAS results of rare variation and psychiatric disorders: ACMG-56 genes. **Table S7.** PheWAS results of rare variation and psychiatric disorders: CACNA1C. **Table S8.** PheWAS results of rare variation and psychiatric disorders: TCF4. **Table S9.** PheWAS of rare variation and psychiatric disorders: interaction with age. **Table S10.** PheWAS of rare variation and psychiatric disorders: interaction with sex. **Table S11.** PheWAS of rare variation and psychiatric disorders: combining variation across 58 genes by functional annotation. **Table S12.** Gene Ontology analysis of the 58 genes: biological process. **Table S13.** Gene Ontology analysis of the 58 genes: cellular component. **Table S14.** Gene Ontology analysis of the 58 genes: molecular function.  **Table S15.** PheWAS of rare variation and psychiatric disorders: 34 genes from the top GO term of biological process. **Table S16.** PheWAS of rare variation and psychiatric disorders: 16 genes from the top GO term of cellular component. **Table S17.** PheWAS of rare variation and psychiatric disorders: 25 genes from the top GO term of molecular function. **Table S18.** Phenotypic comparison between eMERGE and UKB-GeneBass. Table S19. PheWAS results of rare variation and psychiatric disorders in UKB-GeneBass. **Table S20.** Suggested gene associations in eMERGE (Table 1) vs. in UKB-GeneBass  

## Data Availability

The eMERGEseq panel analyzed in this study has been deposited on dbGaP (https://www.ncbi.nlm.nih.gov/gap/) and can be requested via the accession number phs001616.v2.p2. The 1000 Genomes phase 3 dataset can be downloaded at https://www.internationalgenome.org/category/phase-3/.

## Abbreviations

eMERGE: The Electronic Medical Records and Genomics consortium
eMERGEseq: A targeted sequence panel designed within the eMERGE network
ACMG: American College of Medical Genetics and Genomics
RoR: Return of results
EHR: Electronic health records
ICD: International Classification of Disease
GWAS: Genome-wide association studies
PheWAS: Phenome-wide association studies
PheCode: PheWAS code
PCA: Principal component analysis
SNP: Single nucleotide polymorphism
MAF: Minor allele frequency
PTV: Protein-truncating variants
FDR: False discovery rate
