# Supplementary material for: Psychiatric manifestations of rare variation in medically actionable genes: a PheWAS approach
Source: BMC Genomics. 2022 May 19;23:385. doi: 10.1186/s12864-022-08600-x (PMC9121574; doi:10.1186/s12864-022-08600-x)
Supplement: Supplementary file 1 — Additional file 1: Figure S1. Ancestry assignment of study individuals using principal component analyses (PCA). Figure S2. Phenotypic correlation heatmap among the 37 curated PheCodes of common psychiatric phenotypes. Figure S3. No inflation in λGC from single variant association analysis for two selected complex traits. Figure S4. Phenome-wide burden of rare variation in LDLR recapitulated known association with hypercholesterolemia. Figure S5. PheWAS of rare variation in the ACMG-56 genes with psychiatric disorders [file 12864_2022_8600_MOESM1_ESM.docx]

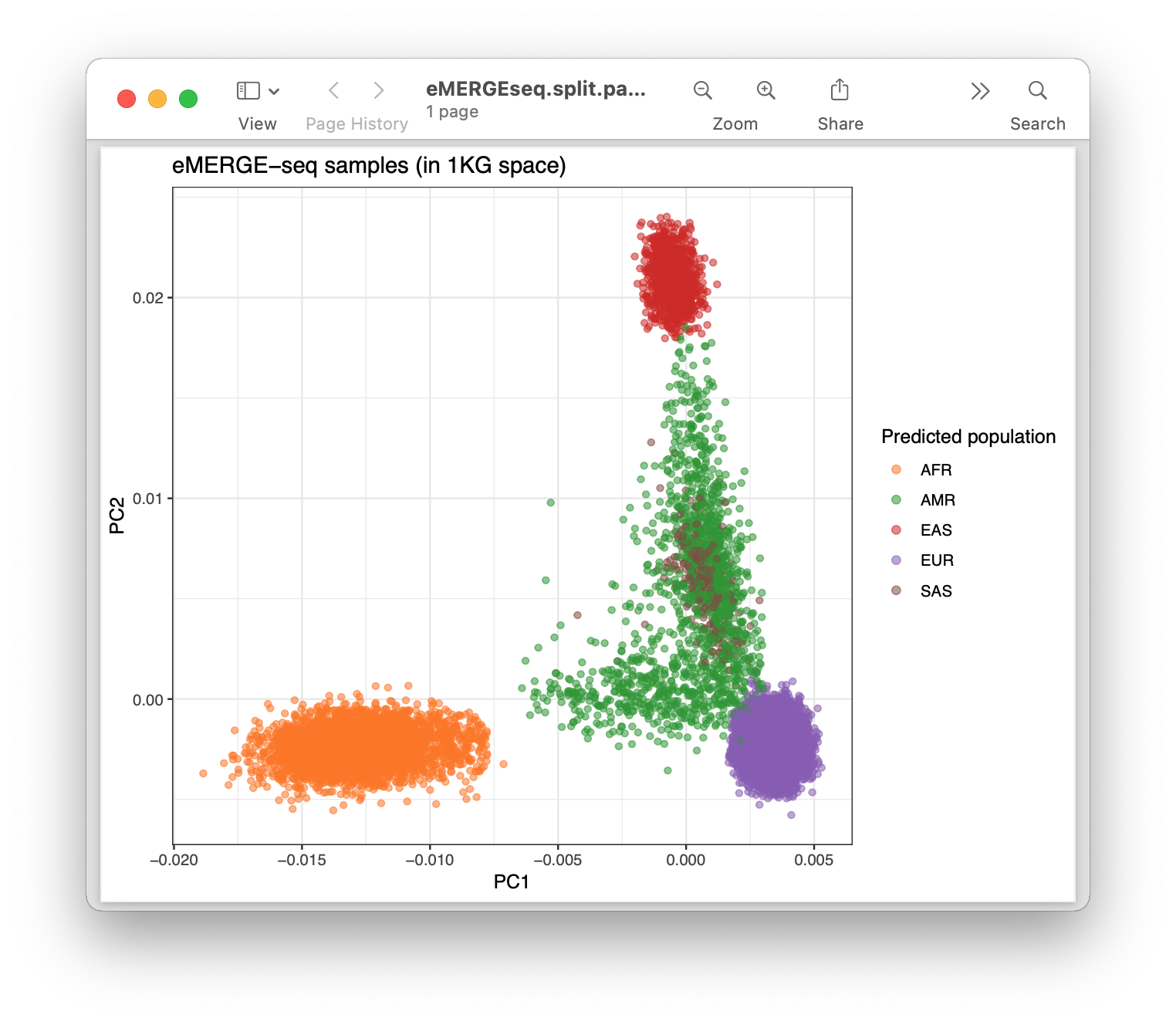


**Figure S1.** Ancestry assignment of study individuals using principal component analyses (PCA)

We performed PCA on study individuals to identify ancestral populations and applied Random Forest Classifier with 1000 Genomes data as the training data to assign ancestry to each individual. With a prediction probability > 0.8, 16,641 participants were classified as European (EUR), 3,612 as African (AFR), 1,455 as Admixed/Latin American (AMR), 1,093 as East Asian (EAS), and 271 as South Asian (SAS), as shown in the figure in distinct clusters. For power considerations, we focused on the EUR subset for subsequent statistical analyses.

**Figure S2.** Phenotypic correlation heatmap among the 37 curated PheCodes of common psychiatric phenotypes

The selected phenotypes have a minimum case count of 75, equivalent to an in-sample prevalence of 0.5%. Pairwise correlation was calculated using Spearman’s correlation coefficient (*ρ*). Hierarchical clustering of the correlation matrix shows clusters of psychiatric disorders based on phenotypic similarity or comorbidity. The highest correlation was observed among “alcoholism” and “alcohol-related disorders” (*ρ* = 0.57), “depression” and “major depressive disorder” (*ρ* = 0.55), “speech and language disorder” and “developmental delays and disorders” (ρ = 0.45), and “autism” and “developmental delays and disorders” (*ρ* = 0.44). The largest negative correlations were observed between “autism” and “tobacco use disorder” (*ρ* = -0.09), “attention deficit hyperactivity disorder” and tobacco use disorder” (*ρ* = -0.07), and “depression” and “autism” (*ρ* = -0.07). More than half of the tested correlations were significant after Bonferroni correction (*P* < 7.5x10^-5^); *non*-significant correlations are labeled with a “cross” sign.

**Figure S3.** No inflation in λ_GC_ from single variant association analysis for two selected complex traits

Panel (A) shows the GWAS results for obesity [Ncase=3521; PheCode: 278.1] for variants with MAF > 0.1%. Panel (B) shows the GWAS results for essential hypertension [Ncase=6972; PheCode: 401.1] for variants with MAF > 0.1%. No inflation is observed in the two GWASes with the genomic inflation factor λ_GC_ ~ 1, suggesting minimal signs of population stratification or other confounding factors in the QC’ed dataset. GWAS for obesity reproduces the established genomic association with the *FTO* locus (A).

**Figure S4.** Phenome-wide burden of rare variation in *LDLR* recapitulated known association with hypercholesterolemia

Using the *LDLR* gene as a positive control, we conducted a PheWAS of rare variant burden analysis across 966 phenotypes with at least 75 cases (in-sample prevalence of 0.5%) in our dataset of 15K individuals via Firth’s logistic regression, adjusting for age, sex, sites, and the first 10 principal components. The results recaptured the established causal relationship between rare deleterious mutations, particularly missense variation, with hypercholesterolemia (labeled on the figure are associations surpassing the Bonferroni-corrected significance of 5.2x10^-5^, as indicated by the vertical red line). Synonymous variation, as a functionally neutral class of variants, showed no inflation in association signals. This test was performed for five categories of qualifying variants separately (from left to right): (1) all variants with MAF < 1%, (2) all non-synonymous variants, (3) all PTVs and damaging missense variants combined, (4) all damaging missense variants, and (5) all PTVs. Each triangle represents a tested trait. Triangles with the tip pointing toward the right indicating an odds ratio (OR) > 1 (risk-increasing), and with the tip pointing toward the left indicating an OR < 1 (risk-decreasing).

**Figure S5. PheWAS of rare variation in the ACMG-56 genes with psychiatric disorders**

For each gene, five-panel results for all 37 tested psychiatric conditions are shown, separately for (1) all variants with MAF < 1%, (2) all non-synonymous variants, (3) all PTVs and damaging missense variants combined, (4) damaging missense variants, and (5) PTVs (from left to right). On the x-axis shows the -log_10_(*p*-value) of the burden tests. Each triangle represents a disorder, with an upright triangle indicating the gene is associated with an increased risk (OR > 1) of the disorder and an inverted triangle indicating a decreased risk (OR < 1). Genes with no qualifying variants present among the study participants were not tested and are left vacant in the figure. The vertical dotted grey line for each individual PheWAS signifies the nominal significance level of 0.05, and the vertical red solid line represents the Bonferroni-corrected significance (0.05/37 = 1.35x10^-3^). No association surpassed the study-wide significance at FDR< 0.05.
